# The Editor's Letter-Box

**Published:** 1916-09-02

**Authors:** John MacKeith

**Affiliations:** To the Secretary, National Health Insurance Commission (England), Buckingham Gate, S.W.,


### THE EDITOR'S LETTER-BOX.

### The Future of Insurance Practice.

*To the Editor of* The Hospital.

Sir,—I enclose copy of a letter I have to-day sent to the National Health Insurance Commission (England), and will be much obliged if you can find room for it in The Hospital.—I am, yours truly, John MacKeith.

15 Cavendish Place, Cavendish Square, W.,

August 29, 1916.

Sir,—I observe that the *Medical World,* which claims to be the "official journal of the P.M.P.U." (Panel Medico-Political Union), in its issues of the 18th and 25th inst., under articles entitled "The Future of Insurance Practice," publishes copies of letters which have been sent, on behalf of the Union, by its General Secretary, Dr. A. Welply, to the Secretary, National Health Insurance Commission (England), and to the Right Hon. Charles Roberts, M.P.

In the concluding paragraph of his letter of the 23rd inst. to the National Health Insurance Commission (England) Dr. Welply finishes up with the following threatening statement: "If the Commissioners decline to accede to this request we shall have no other alternative (and, indeed, it is our duty so to do) but to seek the opinion of our members as to resigning *en bloc* from the panel at the appropriate time."

As they are of such recent date it is unnecessary for me to remind the Commissioners of the events associated with the passing of the National Health Insurance Act, in so far as the medical profession is concerned. It will be recalled, however, that the present Secretary of State for War, Mr. Lloyd George, who was responsible for its introduction, was *threatened* with all manner of terrible consequences to the health of the community if he persisted with the Bill, in the face of the opposition of a practically unanimous profession to its provisions as regards medical "benefits." Dr. J. Smith Whitaker, late Medical Secretary, British Medical Association, and, I believe, at present one of the Commissioners and Deputy Chairman to the National Health Insurance Commission, will be able to inform his co-Commissioners of the actual numbers of the medical profession which the British Medical Association claimed to represent. All the threats of a "unanimous" profession turned out to be mere "bluff." When "threatened" with exclusion for a considerable period if they failed to join the "Panel" by a certain date, a certain type of member of the honourable and noble profession of medicine were tumbling over each other in their haste and anxiety to get their names on the "Panel" in time.

Never again, until the profession has been purged of its "treachery" in tearing up its pledges as so many "scraps of paper," will it be possible to unite the "profession" in its demands for "justice." The "cream" of the profession is represented by those members who have suffered much, and have been prepared to suffer all, pecuniary loss rather than profit by having anything to do with the iniquitous and degrading, although enriching, "Panel" system. The Commissioners need take no notice of the "threat" of the Panel Medico-Political Union. I know many of the medical men on the "Panel" too well, having acted as Honorary Secretary to a Local Medical Committee previous to the passing of the National Health Insurance Act. All and every "insidious" attempts, even if accompanied by the most "discourteous attitude" of which the Commissioners are capable, "to thrust new regulations upon Panel practitioners" will be forgiven, if not forgotten, if only the Panel practitioner is allowed to continue to draw his present rate of pay for "work" which he does in such a perfunctory manner. A State medical service, in which the "cream" of the profession would be willing to participate, is that which the Panel practitioner, generally speaking, is most anxious to prevent being inaugurated.—I am, Sir, etc.,

To the Secretary, John MacKeith, M.B.

National Health Insurance Commission (England),

Buckingham Gate, S.W.,

